# Cell-intrinsic IL-7Rα deficiency in Foxp3^+^ Tregs increases type 1 diabetes incidence in NOD mice

**DOI:** 10.1093/immhor/vlag034

**Published:** 2026-08-04

**Authors:** Albert R Jones IV, Peyton S Smith, Hans Dooms

**Affiliations:** Arthritis and Autoimmune Diseases Center, Rheumatology Section, Department of Medicine, Boston University Chobanian & Avedisian School of Medicine, Boston, MA, United States; Department of Virology, Immunology & Microbiology, Boston University Chobanian & Avedisian School of Medicine, Boston, MA, United States; Department of Immunology and Genomic Medicine, National Jewish Health, Denver, CO, United States; Arthritis and Autoimmune Diseases Center, Rheumatology Section, Department of Medicine, Boston University Chobanian & Avedisian School of Medicine, Boston, MA, United States; Department of Virology, Immunology & Microbiology, Boston University Chobanian & Avedisian School of Medicine, Boston, MA, United States; Department of Immunology and Genomic Medicine, National Jewish Health, Denver, CO, United States; Department of Immunology and Microbiology, University of Colorado Anschutz Medical Campus, Aurora, CO, United States

**Keywords:** Interleukin-7 receptor, Tregs, type 1 diabetes

## Abstract

Regulatory CD4^+^ T cells (Tregs), as defined by expression of the transcription factor Foxp3, strongly depend on the cytokine interleukin 2 (IL-2) for their survival and function and are often identified by the combination of high IL-2Rα (CD25) and low IL-7Rα (CD127) expression. Nevertheless, subsets expressing higher levels of IL-7Rα have been described, and IL-7 signaling does play a role in Treg function in some specific biologic contexts and tissues. The precise role of IL-7Rα-expressing Tregs in autoimmunity remains poorly defined though, potentially hampering current efforts to develop IL-7Rα blockade for the treatment of various autoimmune diseases. To ask whether cell-intrinsic IL-7Rα expression in Tregs was required for their function during type 1 diabetes (T1D) development, we generated *non-obese* diabetic (NOD) mice in which IL-7Rα is exclusively deleted in Foxp3-expressing Tregs. In young NOD mice, IL-7Rα deficiency did not alter Treg numbers and phenotype. However, 100% of NOD mice with IL-7Rα-deficient Tregs became diabetic, while the T1D incidence is typically around 50–60% in our colony. This increased susceptibility for T1D indicates that IL-7Rα expression in Tregs is required to protect a subset of NOD mice against islet autoimmunity. At the time of T1D onset, CD4^+^ and CD8^+^ T cells from NOD mice with IL-7Rα-deficient Tregs showed increased IFN-γ and IL-2 cytokine production. Our data demonstrate that cell-intrinsic IL-7R signaling in Foxp3^+^ Tregs is required to suppress effector T cell responses and to prevent full penetrance of T1D in NOD mice.

## Introduction

Foxp3^+^ Tregs play critical roles in maintaining self-tolerance by suppressing autoimmune responses. Tregs are strongly dependent on signals from the γc cytokine IL-2 for their survival and function,^1^ and suboptimal functioning of the IL-2 pathway, leading to defective Tregs, has been shown to contribute to autoimmune disease development, including T1D.^2–4^ Foxp3^+^ Tregs are often identified by high expression of IL-2Rα and low expression of IL-7Rα, which distinguishes these cells from “conventional” CD4^+^ T cells that express low levels of IL-2Rα and high IL-7Rα in the naive and memory state.^5^ This opposite phenotype and dependence on the respective cytokines is being leveraged for treatment of autoimmune diseases. We and others have shown that blocking IL-7Rα reduces the functionality of autoreactive T cells in T1D, presumably without significantly affecting Tregs that are critical to preserve in the setting of autoimmune disease.^6^^,^^7^ Although low IL-7Rα expression suggests that IL-7 is not a major cytokine in Treg biology, some studies have nevertheless revealed specific situations where IL-7Rα is upregulated and IL-7 signaling is contributing to the survival/maintenance of Treg populations in some tissues.^8–11^ For example, memory Tregs in the skin were reduced after treatment of mice with anti-IL-7Rα monoclonal antibodies (mAbs),^12^ and Tregs with a specific deletion of IL-7Rα showed reduced capacity to protect skin allografts from rejection,^9^ while intragraft IL-7Rα^high^ Tregs maintained transplant tolerance. C57BL/6 mice with a Foxp3-Cre-driven deletion of IL-7Rα showed a modest reduction of Tregs with a CD62^low^ CD44^hi^ effector phenotype and developed mild lung and liver autoimmunity at older age.^9^ Anti-CD3 therapy of T1D was also shown to induce IL-7Rα-dependent Treg populations.^13^^,^^14^ These studies underscore the need to understand the role of cell-intrinsic IL-7 signaling in Foxp3^+^ Tregs to better tailor future use of IL-7 targeting for therapy of autoimmune diseases.

To accurately define the cell-intrinsic role of IL-7Rα in Tregs during T1D development, we generated NOD mice in which IL-7Rα is deleted exclusively in Foxp3^+^ Tregs. We found that Treg numbers were similar in pre-diabetic and new-onset NOD mice with IL-7Rα-deficient vs -sufficient Tregs, indicating that cell-intrinsic IL-7Rα was not required for Treg homeostasis. Importantly, however, our colony of female NOD mice with IL-7Rα-deficient Tregs showed full T1D penetrance, while a fraction of the mice in control cohorts remained diabetes-free. Restimulation of splenocytes from diabetic mice revealed increased cytokine responses in cultures from mice with IL-7Rα-deficient Tregs, further suggesting a functional defect in the absence of IL-7Rα. Thus, although IL-7Rα deficiency in Tregs does not lead to major losses of Treg numbers, specific functions related to controlling the development and/or function of IFN-γ-producing T cells appear to be impacted, reducing protection against T1D in NOD mice. These findings have implications for immunotherapies using Tregs or targeting the IL-7 pathway.

## Materials and methods

### Ethics statement

All animal work including handling, breeding, euthanasia and tissue harvesting have been reviewed and approved by Boston University Chobanian & Avedisian School of Medicine’s Institutional Care and Use Committee (IACUC) (protocol number: PROTO201800235), and the National Jewish Health IACUC (protocol number: 2022-0001). Protocols followed the Guide for the Care and Use of Laboratory Animals and experiments were designed to minimize animal pain and suffering.

### Mice

NOD.IL7Rα^fl/fl^ mice were generated by backcrossing C57Bl/6 IL-7Rα^fl/fl^ (Il7rtm1.1Asin/J, Jackson cat. no. 022143) mice for 12 generations on the NOD background. NOD.Foxp3-EGFP/Cre × IL-7Rα^fl/fl^ mice were generated in our laboratory by breeding the backcrossed NOD.IL-7Rα^fl/fl^ mice with NOD.Foxp3-EGFP/Cre mice (NOD/ShiLt-Tg (Foxp3-EGFP/Cre)1cJbs/J, Jackson cat. no. 008694). Presence of the IL-7Rα^fl/fl^ and Foxp3-EGFP/Cre alleles was confirmed using PCR.

### Blood glucose measurements

Blood glucose measurements were taken weekly starting when mice reached 8–12 weeks of age. Measurements were taken using a Contour blood glucose reader. Mice were considered diabetic when there were 2 consecutive blood glucose readings ≥ 250 mg/dL.

### Tissue harvest

Spleens and pancreatic lymph nodes (PLN) were isolated and single-cell suspensions prepared using a 40 µM tissue strainer (Fisher). Cells were washed twice with PBS and splenocytes incubated in ACK lysis buffer (Invitrogen) for 3 min at room temperature to lyse red blood cells. Pancreata were incubated for 30 min at 37 °C in collagenase P (1 mg/mL; Roche), DNase (20 μg/mL) and 0.2% BSA in RPMI medium. Digested tissue was strained through a 70 μM cell strainer and washed once with RPMI medium containing DNase. Lymphocytes were enriched using a 40%/60% Percoll gradient.

### Flow cytometry and antibodies

For flow cytometry staining, cells were washed twice in 1× PBS, counted and stained with Live/Dead stain for 30 min in the dark at 4 °C. Cells were then washed twice with 1× PBS, 2% FBS and re-suspended in Fc block buffer for 5 min. A master mix containing fluorescent antibodies for cell surface markers was added to each sample and incubated for 15 min in the dark at 4 °C. Cells were then washed twice with 1× PBS, 2% FBS, re-suspended in Foxp3Fix/Perm (Invitrogen) and left at 4 °C overnight. The following day, cells were washed twice with permeabilization (Invitrogen) buffer and then re-suspended in permeabilization buffer containing fluorescent antibodies for intracellular targets and left at 4 °C for 30 min. Cells were washed with permeabilization buffer and re-suspended in 1× PBS, 2% FBS. Samples were analyzed using a BD LSRII or BD Fortessa flow cytometer. The following reagents and antibodies were used: Live/Dead Fixable Yellow or Aqua (ThermoFisher), FITC, PerCP, APC-e780, or PE-e610 rat anti-mouse CD4 (clone RM4-5, BD or Invitrogen), PercP rat anti-mouse CD8a (clone 53-6.7, Invitrogen), biotin-conjugated anti-mouse CD127 (clone A7R34, Invitrogen), Streptavidin-/PE (eBioscience), APC rat anti-mouse CD25 (clone PC61, Biolegend), PE rat anti-mouse PD-1 (clone RMP1-30, Invitrogen), PE-Cy7 rat anti-mouse TIGIT (clone GIGD7, Invitrogen), FITC or e450 rat anti-mouse/rat Foxp3 (clone FJK-16s, Invitrogen), PE or PE-Cy7 rat anti-mouse IFN-γ (clone XMG1.2, BD), and PE-Cy7 or e450 rat anti-mouse IL-2 (clone JES6-5H4, Biolegend, Invitrogen).

### In vitro stimulation for intracellular cytokine staining

Splenocytes, isolated as described above, were counted and plated at 1 × 10^6^ cells per well in a 24-well plate in RPMI 1640 medium with 1 mM non-essential amino acids, 1 mM sodium pyruvate, 55 nM β-mercaptoethanol, 10 mM HEPES, 100 μg/mL penicillin/streptomycin, and 10% FBS (Omega Scientific). Samples from each mouse were plated in 2 wells: one unstimulated control and one treated with 20 ng/mL of phorbol myristate acetate (PMA) and 0.5 μg/mL of Ionomycin. After 1 h, Brefeldin A, a protein transport inhibitor, was added to the cultures to trap cytokines inside the cells. Cells were then incubated at 37 °C with 5% CO_2_ for 4–6 h. After the incubation period, cells were washed with PBS and intracellularly stained using the flow cytometry protocol detailed above.

### Statistics

All statistics were performed using Prism software. Statistically significant differences between groups were determined using the Log-rank (Mantel-Cox) test for diabetes incidence, *t* tests and 2-way ANOVA with multiple comparisons (uncorrected Fisher’s LSD) for phenotypic data, and mixed-effects 2-way ANOVA with Sidak’s multiple comparison tests for cytokine data.

## Results

**Figure 1:**
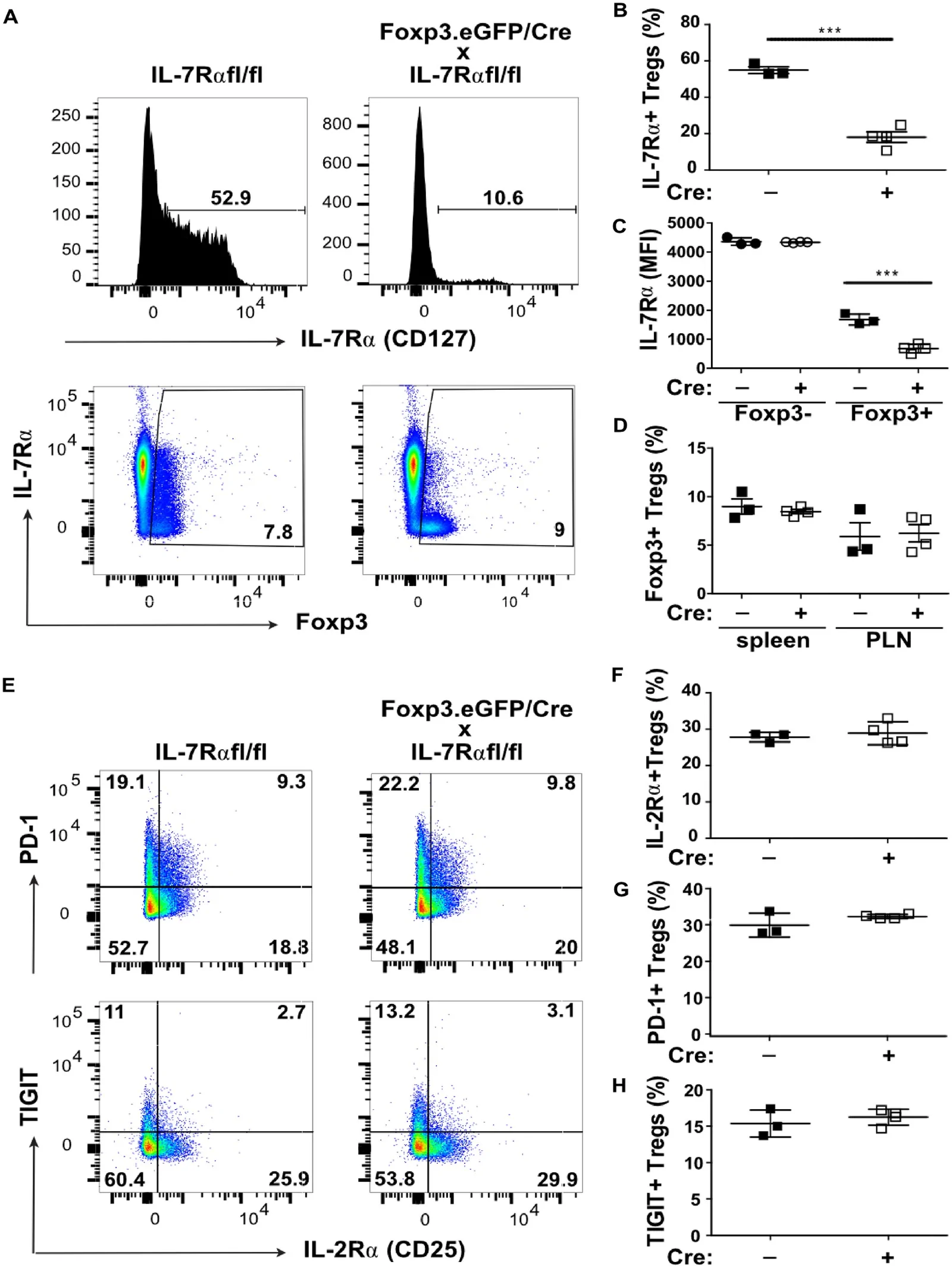
Cell-intrinsic loss of IL-7Rα expression does not alter the frequency and phenotype of Foxp3^+^ Tregs in young NOD mice. PLNs and spleens were harvested from 4-wk old NOD.IL-7Rα^fl/fl^ and NOD.Foxp3.eGFP/Cre × IL-7Rα^fl/fl^ mice and cell suspensions stained with fluorescently-labeled mAbs to detect Foxp3, IL-7Rα, CD25, PD-1 and TIGIT by flow cytometry. (A) Upper panels: Representative histograms show IL-7Rα expression in Foxp3^+^ Tregs from NOD.IL-7Rα^fl/fl^ (Cre-) and NOD.Foxp3.eGFP/Cre × IL-7Rα^fl/fl^ (Cre+) mice. Lower panels: Representative dot plots show frequencies (%) of Foxp3^+^ Tregs within the CD4^+^ T cell gate. Summary graphs show percentages of (B) IL-7Rα-expressing cells within the CD4^+^ Foxp3^+^ gate, (C) IL-7Rα expression levels (MFI) in Foxp3^neg^ vs Foxp3^+^ CD4^+^ T cells from both mouse strains (*n* = 3–4), and (D) Foxp3^+^ cells within CD4^+^ T cells (*n* = 3–4). (E) Representative dot plots show PD-1 and TIGIT expression vs CD25 expression gated on CD4^+^ T cells. Graphs show the percentages of (F) CD25^+^, (G) PD-1^+^, and (H) TIGIT^+^ cells within the CD4^+^Foxp3^+^ Treg gate from NOD.IL-7Rα^fl/fl^ (*n* = 3) and NOD.Foxp3.eGFP/Cre × IL-7Rα^fl/fl^ (*n* = 4) mice. *** = *P* < 0.001.

To study the role of IL-7 signaling in Tregs during an ongoing autoimmune disease, we backcrossed IL-7Rα^fl/fl^ mice onto the NOD background and subsequently crossed this strain with NOD.Foxp3-EGFP/Cre mice to generate NOD mice in which IL-7Rα was deleted in Foxp3-expressing Tregs only. Cre-driven deletion of the IL-7Rα was effective and specific: while >50% of Foxp3^+^ Tregs expressed IL-7Rα in the Cre- IL-7Rα^fl/fl^ mice, less than 20% IL-7Rα^+^ Tregs were detected in Cre-expressing mice (Fig. 1A, B). Confirming specificity, IL-7Rα expression in CD4^+^ conventional T cells was normal (Fig. 1A, C). Next, we assessed whether IL-7Rα-deficiency in Foxp3^+^ Tregs led to changes in their numbers and/or phenotype in young, pre-diabetic mice. We found that Treg frequencies were similar in the spleens and pancreatic lymph nodes (PLN) of 4-wk old mice (Fig. 1A, D). Moreover, expression of IL-2Rα (CD25), a canonical marker and critical receptor for Treg function, remained unaffected (Fig. 1E, F). Note that IL-2Rα expression on Tregs from NOD mice is low compared to other mouse strains, as has been described.^15^^,^^16^ We previously showed that treatment of NOD mice with anti-IL-7Rα blocking mAbs increased expression of the co-inhibitory receptor (co-IR) PD-1 on effector T cells as well as Tregs.^6^^,^^17^ Although anti-IL-7Rα mAb treatment of NOD mice was overall protective against T1D, PD-1 expression on Tregs has been shown to reduce their capacity to suppress the anti-islet response,^18^ emphasizing the importance of understanding the mechanisms of PD-1 regulation in Foxp3^+^ Tregs. However, systemic blockade of IL-7Rα by anti-IL-7Rα mAbs does not allow to determine whether co-IR expression in Tregs is regulated by cell-intrinsic IL-7 signaling or is a consequence of broad changes in the immune system. Therefore, to definitively establish whether absence of IL-7 signaling in Foxp3 Tregs directly leads to enhanced co-IR expression, we measured PD-1 and TIGIT levels in IL-7Rα-deficient vs -sufficient Tregs. We found no difference at 4 weeks of age (Fig. 1E, G, H). Thus, we conclude that IL-7Rα deficiency does not impede development and early peripheral survival and fitness of Foxp3^+^ Tregs in NOD mice.

**Figure 2:**
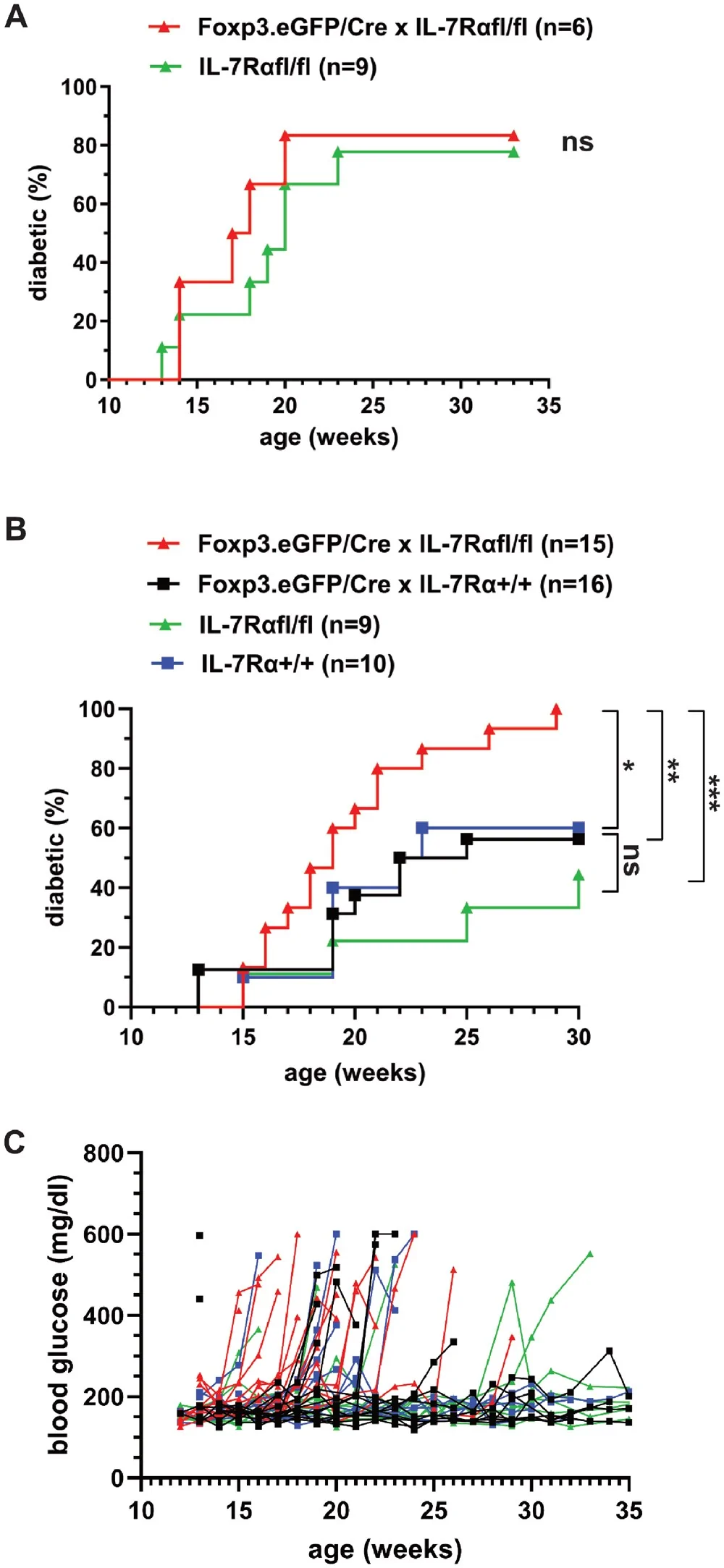
NOD mice with IL-7Rα-deficient Foxp3^+^ Tregs show increased T1D incidence. Blood glucose levels were measured weekly in female NOD.IL-7Rα^fl/fl^ (green triangles), NOD.Foxp3.eGFP/Cre × IL-7Rα^fl/fl^ (red triangles), NOD.Foxp3.eGFP/Cre × IL-7Rα^+/+^ (black squares), and NOD.IL-7Rα^+/+^ (blue squares) mice starting at 8 or 12 wks of age. Mice were considered diabetic after two consecutive blood glucose measurements ≥ 250 mg/dL. Kaplan–Meier plots show diabetes incidence in the indicated strains at (A) Boston University Medical Campus, and (B) National Jewish Health. (C) Graph shows weekly blood glucose values from the indicated strains at National Jewish Health. * = *P* < 0.05, ** = *P* < 0.01, *** = *P* < 001.

Despite the lack of obvious differences in Treg phenotype in young Foxp3-EGPP/Cre × IL-7Rα^fl/fl^ mice, it was feasible that chronic absence of IL-7 signaling in Tregs would impact the course of T1D development. Thus, we assessed the impact of Treg-specific IL-7Rα deletion on T1D incidence by following cohorts of NOD.Foxp3-EGFP/Cre × IL-7Rα^fl/fl^ and NOD.IL-7Rα^fl/fl^ mice by weekly measurements of urine glucose levels at the Boston University Medical Campus (BUMC) animal facility. In mice that showed elevated urine glucose, hyperglycemia and diabetes onset was confirmed by blood glucose measurements. NOD mice with IL-7Rα-deficient Tregs showed a slightly earlier median time of T1D onset than controls: 17.5 wks vs 20 wks, respectively (Fig. 2A). However, this difference did not reach statistical significance due to the small cohorts followed in this study, and the already high T1D incidence in the control group at the BUMC facility. We performed a more comprehensive study at the National Jewish Health (NJH) animal facility, comparing larger cohorts of 4 different genotypes, namely NOD.Foxp3-EGFP/Cre × IL-7Rα^fl/fl^, NOD.Foxp3-EGFP/Cre × IL-7Rα^+/+^, NOD.IL-7Rα^fl/fl^, and wild type (IL-7Rα^+/+^) NOD mice. Importantly, we found that NOD mice with Treg-specific IL-7Rα deficiency were significantly more susceptible to T1D development. In fact, 100% of females developed T1D while the average for all 3 control genotypes was around 50%–60% (Fig. 2B), as revealed by blood glucose measurements (Fig. 2C). Incidence for T1D can significantly differ between institutions and animal facilities due to environmental factors (e.g. microbiome, pathogens),^19^^,^^20^ and hence the lower penetrance in the control groups at NJH further unmasked the protective role of IL-7 signaling in NOD Foxp3^+^ Tregs. We conclude that at least the fraction of NOD mice that typically does not become diabetic is dependent on IL-7Rα-expressing Tregs for protection against islet autoimmunity.

**Figure 3:**
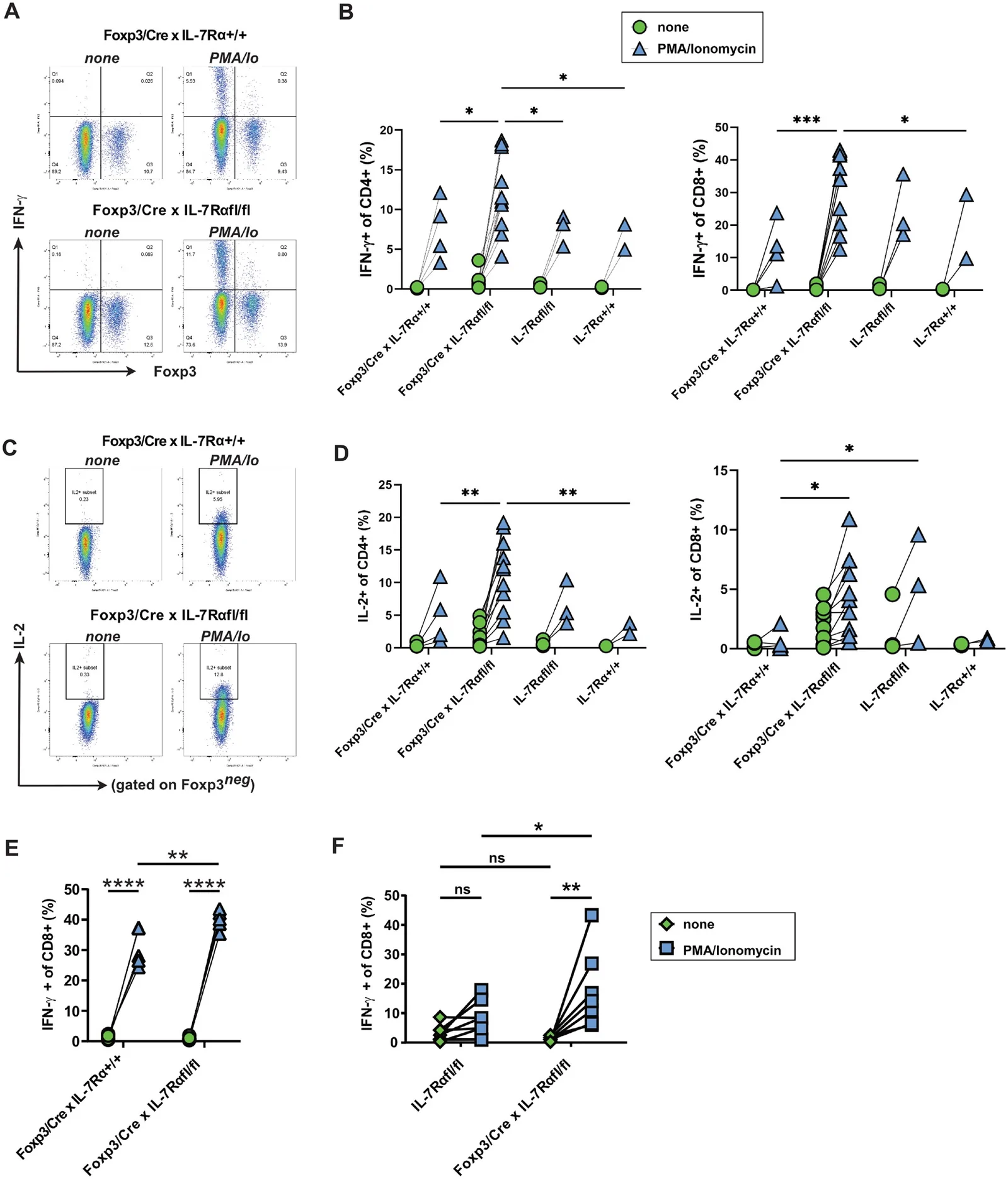
Conventional T cells from NOD mice with IL-7Rα-deficient Foxp3^+^ Tregs show increased cytokine production. Splenocytes from NOD.IL-7Rα^fl/fl^, NOD.Foxp3.eGFP/Cre × IL-7Rα^fl/fl^, NOD.Foxp3.eGFP/Cre × IL-7Rα^+/+^, and NOD.IL-7Rα^+/+^ mice were isolated and stimulated *ex vivo* with PMA and Ionomycin. Brefeldin A was added after 1 hr. After 4–6 hrs, cultures were harvested and intracellular cytokine staining performed for flow cytometry. (A) Representative dot plots of IFN-γ and Foxp3 staining in CD4^+^ T cells, (B) Summary graphs show frequencies (%) of IFN-γ^+^ cells within the CD4^+^ Foxp3^neg^ (left) and CD8^+^ (right) gates, (C) Representative dot plots of IL-2 staining in CD4^+^ Foxp3^neg^ T cells, (D) Summary graphs show frequencies (%) of IL-2^+^ cells within the CD4^+^ Foxp3^neg^ (left) and CD8^+^ (right) gates. All data are from the indicated new-onset diabetic strains at National Jewish Health. (E) Frequencies (%) of IFN-γ^+^ CD8^+^ T cells from pre-diabetic mice (9 wks old) at National Jewish Health. (F) Frequencies (%) of IFN-γ^+^ CD8^+^ T cells from new-onset diabetic mice at Boston University Medical Campus. Lines connect control vs stimulated splenocyte cultures from each individual mouse. * = *P* < 0.05, ** = *P* < 0.01, *** = *P* < 0.001, **** = *P* < 0.0001.

To assess whether the reduced protective capacity of IL-7Rα-deficient Tregs was reflected in the state of effector T cells at T1D onset, we restimulated splenocytes with PMA and ionomycin *ex vivo* and assessed their capacity to produce cytokines by intracellular cytokine staining (Fig. 3). We found that CD4^+^ and CD8^+^ T cells from NOD.Foxp3-EGFP/Cre × IL-7Rα^fl/fl^ mice showed significantly increased IFN-γ (Fig. 3A, B) and IL-2 (Fig. 3C, D) production after stimulation, compared to cells from IL-7Rα-sufficient control groups. Interestingly, increases in IFN-γ-producing CD8^+^ T cells were already present in pre-diabetic NOD.Foxp3-EGFP/Cre × IL-7Rα^fl/fl^ mice (9 wks old) (Fig. 3E). CD4^+^ T cells did not show increased IFN-γ production in these younger mice (data not shown). Finally, we also observed increased IFN-γ production in CD8^+^ T cells from Treg-specific IL-7Rα-deficient NOD mice at the BUMC facility (Fig. 3F), further underscoring the robustness and reproducibility of this finding. Hence, although no significant increases in overall numbers of conventional T cells were found in NOD.Foxp3-EGFP/Cre × IL-7Rα^fl/fl^ mice (data not shown), IL-7Rα deficiency in Tregs leads to a less suppressive immune environment with increased effector T cell activation and/or differentiation.

To formally confirm that Treg frequency and phenotype remained unaffected by the absence of IL-7 signaling at the time of hyperglycemia onset, we determined their numbers, as well as IL-2Rα, PD-1 and TIGIT expression in spleen, PLN and pancreas of newly diabetic mice from the various genotypes. In the colony at NJH, no statistically significant differences were observed in Foxp3^+^ Treg frequencies and absolute numbers, nor in IL-2Rα, PD-1 and TIGIT expression (data not shown). Analysis of the Tregs from new-onset diabetic mice at the BUMC facility did show some modest differences in the frequencies and phenotypes of IL-7Rα-deficient Tregs, notably a minor reduction in splenic Tregs and higher expression of PD-1 and TIGIT (data not shown). This is likely due to environmental differences between the facilities (e.g. microbiome, pathogens) but, since this phenotype was variable, it cannot explain the increased T1D incidence and IFN-γ production in NOD mice with IL-7Rα-deficient Tregs.

## Discussion

The IL-7 signaling pathway is an attractive target to treat autoimmune diseases due to its well-documented role in maintaining functional memory T cells while at the same time appearing to be less critical for the survival and function of Foxp3^+^ Tregs, which protect against autoimmunity.^21^ In various animal models of autoimmune disease, including T1D, treatment with anti-IL-7Rα mAbs that block IL-7 signaling led to improved outcomes. A Phase I clinical trial in T1D patients reduced the numbers and activity of memory T cells while largely preserving naive T cells and Tregs in the blood. Although this immunomodulatory activity supported the promise of blocking IL-7Rα in T1D, initial analysis of C-peptide levels did not indicate a clinical response, albeit the study was not powered for this purpose.^22^ Although Foxp3^+^ Tregs are mainly dependent on IL-2 for their survival and functions, including in T1D,^2^^,^^23^ there are nevertheless studies indicating that IL-7 signaling in Tregs is required under specific circumstances. For example, Tregs that were capable of protecting against diabetes in NOD mice expressed high levels of IL-7Rα and were dependent on IL-7.^8^^,^^13^^,^^14^ In normal mice, deletion of IL-7Rα in Foxp3^+^ Tregs led to slow development of mild autoimmunity.^9^ Tregs expressing high levels of IL-7Rα have been identified in the skin, the bone marrow and under lymphopenic conditions.^10–12^ Hence it cannot be excluded that systemic IL-7Rα blockade interferes with the functioning of these IL-7-dependent Treg subsets, which may offset some of the therapeutic efficacy of IL-7 blockade in autoimmunity. It is therefore important to clearly define the role of cell-intrinsic IL-7Rα in Foxp3^+^ Tregs during the development of autoimmune disease. Here we approached this problem by generating NOD mice in which only Foxp3^+^ Tregs are deficient in IL-7Rα. In a comprehensive study we performed at NJH, we found that T1D incidence was increased to 100% compared to 50%–60% in NOD strains with IL-7Rα-sufficient Foxp3^+^ Tregs. However, it is noteworthy that in our earlier study at BUMC, this difference was obscured by overall higher incidence rates in the NOD colony. Thus IL-7R signaling in Foxp3^+^ Tregs protects the fraction of female NOD mice that typically do not become diabetic. Analysis of Treg numbers and phenotypes revealed that IL-7Rα-deficient Tregs appeared normal in young mice. Interestingly however, in the later stages of disease, NOD mice with IL-7Rα-deficient Tregs showed increased IFN-γ- and IL-2-producing CD4^+^ and CD8^+^ T cells. Since mice with IL-7Rα-deficient Tregs did not show broad autoimmunity or lymphoproliferation, our data suggest that IL-7 signaling in Tregs is required for very specific regulatory functions related to controlling cytokine production in effector T cells.

Our previous studies treating NOD mice systemically with anti-IL-7Rα mAbs revealed increased PD-1 expression in Foxp3^+^ Tregs,^6^^,^^17^ potentially compromising their suppressive function in T1D.^18^ However, we found that increased PD-1 expression in IL-7Rα-deficient Tregs was not consistent across facilities. Similarly, reductions in Treg frequencies and IL-2Rα expression were highly variable, and, therefore, we do not consider these site-specific changes a credible mechanistic explanation for the increased T1D susceptibility of NOD.Foxp3-EGFP/Cre × IL-7Rα^fl/fl^ mice. The observation that the exhaustion phenotype was not replicated in IL-7Rα-deficient Tregs after relocation of the mouse colony from BUMC to NJH, but increased IFN-γ production was consistent between facilities, raises important considerations regarding robustness and reproducibility of immune phenotypes in pre-clinical models. Our data support the notion that multi-laboratory studies may increase replicability of discoveries by more reliably identifying the most robust phenotypes, while generalizing findings from single facilities with each their own strictly controlled, specific and standardized conditions, carries risks of overinterpretation. Recent studies aimed at addressing the underlying causes for current concerns about reproducibility in animal experiments have revealed that the rearing environment is a critical modulator of mouse phenotypes, strong enough to influence epigenetic features.^24^ Thus, our study contributes to the ongoing discussion about the need for environmental standardization vs multi-laboratory approaches to achieve higher reproducibility and avoid idiosyncratic phenotypes in animal experiments.^25^

Although we and others previously showed that systemic anti-IL-7Rα blockade in NOD mice prevented and reversed T1D,^6^^,^^7^ indicating that the inhibitory effect on effector/memory T cells dominated, our current data suggest that caution is necessary when treating autoimmune diseases in humans with anti-IL-7Rα mAbs. The compromised functionality of Foxp3^+^ Tregs deprived of IL-7 signals may reduce treatment efficacy in specific biologic contexts during progressive autoimmune disease, or in specific patient populations.

## Data Availability

The data underlying this article will be shared on reasonable request to the corresponding author.

## References

[1] Abbas AK , Trotta E, R Simeonov D, Marson A, Bluestone JA. Revisiting IL-2: biology and therapeutic prospects. Sci Immunol. 2018;3:eaat1482. 10.1126/sciimmunol.aa1482 29980618

[2] Tang Q et al Central role of defective interleukin-2 production in the triggering of islet autoimmune destruction. Immunity. 2008;28:687–697. 10.1016/j.immuni.2008.03.016 PMC2394854 18468463

[3] Gupta S , Cerosaletti K, Long SA. Renegade homeostatic cytokine responses in T1D: drivers of regulatory/effector T cell imbalance. Clin Immunol. 2014;151:146–154. 10.1016/j.clim.2014.02.007 24576418

[4] Hulme MA , Wasserfall CH, Atkinson MA, Brusko TM. Central role for interleukin-2 in type 1 diabetes. Diabetes. 2012;61:14–22. 10.2337/db11-1213 PMC3237657 22187370

[5] Liu W et al CD127 expression inversely correlates with FoxP3 and suppressive function of human CD4+ T reg cells. J Exp Med. 2006;203:1701–1711. 10.1084/jem.20060772 PMC2118339 16818678

[6] Penaranda C et al IL-7 receptor blockade reverses autoimmune diabetes by promoting inhibition of effector/memory T cells. Proc Natl Acad Sci U S A 2012;109:12668–12673. 10.1073/pnas.1203692109 PMC3411948 22733744

[7] Lee LF et al Anti-IL-7 receptor-alpha reverses established type 1 diabetes in nonobese diabetic mice by modulating effector T-cell function. Proc Natl Acad Sci U S A. 2012;109:12674–12679. 10.1073/pnas.1203795109 PMC3412026 22733769

[8] Li CR , Deiro MF, Godebu E, Bradley LM. IL-7 uniquely maintains FoxP3(+) adaptive Treg cells that reverse diabetes in NOD mice via integrin-beta7-dependent localization. J Autoimmun. 2011;37:217–227. 10.1016/j.jaut.2011.06.002 PMC3431214 21745722

[9] Schmaler M et al IL-7R signaling in regulatory T cells maintains peripheral and allograft tolerance in mice. Proc Natl Acad Sci U S A. 2015;112:13330–13335. 10.1073/pnas.1510045112 PMC4629352 26450881

[10] Simonetta F et al Increased CD127 expression on activated FOXP3+CD4+ regulatory T cells. Eur J Immunol. 2010;40:2528–2538. 10.1002/eji.201040531 20690182

[11] Simonetta F et al Interleukin-7 influences FOXP3+CD4+ regulatory T cells peripheral homeostasis. PLoS One. 2012;7:e36596. 10.1371/journal.pone.0036596 PMC3346843 22586481

[12] Gratz IK et al Cutting Edge: memory regulatory T cells require IL-7 and not IL-2 for their maintenance in peripheral tissues. J Immunol. 2013;190:4483–4487. 10.4049/jimmunol.1300212 PMC3660612 23543753

[13] Nishio J , Feuerer M, Wong J, Mathis D, Benoist C. Anti-CD3 therapy permits regulatory T cells to surmount T cell receptor-specified peripheral niche constraints. J Exp Med. 2010;207:1879–1889. 10.1084/jem.20100205 PMC2931163 20679403

[14] Li L , Nishio J, van Maurik A, Mathis D, Benoist C. Differential response of regulatory and conventional CD4(+) lymphocytes to CD3 engagement: clues to a possible mechanism of anti-CD3 action? J Immunol. 2013;191:3694–3704. 10.4049/jimmunol.1300408 PMC3932531 23986534

[15] Godoy GJ et al T regulatory cells from non-obese diabetic mice show low responsiveness to IL-2 stimulation and exhibit differential expression of anergy-related and ubiquitination factors. Front Immunol. 2019;10:2665. 10.3389/fimmu.2019.02665 PMC6886461 31824482

[16] Godoy GJ et al Differences in T regulatory cells between mouse strains frequently used in immunological research: Treg cell quantities and subpopulations in NOD, B6 and BALB/c mice. Immunol Lett. 2020;223:17–25. 10.1016/j.imlet.2020.04.006 32330480

[17] Vazquez-Mateo C , Collins J, Fleury M, Dooms H. Broad induction of immunoregulatory mechanisms after a short course of anti-IL-7Ralpha antibodies in NOD mice. BMC Immunol. 2017;18:18. 10.1186/s12865-017-0201-4 PMC5372316 28356069

[18] Tan CL et al PD-1 restraint of regulatory T cell suppressive activity is critical for immune tolerance. J Exp Med. 2021;218:e20182232. 10.1084/jem.20182232 PMC7543091 33045061

[19] Chen D , Thayer TC, Wen L, Wong FS. Mouse models of autoimmune diabetes: the nonobese diabetic (NOD) mouse. Methods Mol Biol. 2020;2128:87–92. 10.1007/978-1-0716-0385-7_6 PMC8253669 32180187

[20] Pearson JA , Wong FS, Wen L. The importance of the Non Obese Diabetic (NOD) mouse model in autoimmune diabetes. J Autoimmun. 2016;66:76–88. 10.1016/j.jaut.2015.08.019 PMC4765310 26403950

[21] Dooms H. Interleukin-7: fuel for the autoimmune attack. J Autoimmun. 2013;45:40–48. 10.1016/j.jaut.2013.06.007 23831438

[22] Herold KC et al Immunomodulatory activity of humanized anti–IL-7R monoclonal antibody RN168 in subjects with type 1 diabetes. JCI Insight. 2019;4:10.1172/jci.insight.126054 PMC6975260 31852846

[23] Barron L et al Cutting edge: mechanisms of IL-2-dependent maintenance of functional regulatory T cells. J Immunol. 2010;185:6426–6430. 10.4049/jimmunol.0903940 PMC3059533 21037099

[24] Jaric I et al The rearing environment persistently modulates mouse phenotypes from the molecular to the behavioural level. PLoS Biol. 2022;20:e3001837. 10.1371/journal.pbio.3001837 PMC9629646 36269766

[25] Richter SH , Garner JP, Wurbel H. Environmental standardization: cure or cause of poor reproducibility in animal experiments? Nat Methods. 2009;6:257–261. 10.1038/nmeth.1312 19333241

